# Diabetes accelerates retinal ganglion cell dysfunction in mice lacking sigma receptor 1

**Published:** 2012-11-30

**Authors:** Yonju Ha, Alan Saul, Amany Tawfik, Eric P. Zorrilla, Vadivel Ganapathy, Sylvia B. Smith

**Affiliations:** 1 Department of Cellular Biology and Anatomy, Medical College of Georgia, Georgia Health Sciences University, Augusta, GA; 2 Vision Discovery Institute, Medical College of Georgia, Georgia Health Sciences University, Augusta, GA; 3 Department of Ophthalmology, Medical College of Georgia, Georgia Health Sciences University, Augusta, GA; 4 CNAD/MIND, The Scripps Research Institute, La Jolla, CA; 5 Department of Biochemistry and Molecular Biology, Medical College of Georgia, Georgia Health Sciences University, Augusta, GA

## Abstract

**Purpose:**

Sigma receptor 1 (σR1) is a non-opioid transmembrane protein that may act as a molecular chaperone at the endoplasmic reticulum–mitochondrial membrane. Ligands for σR1, such as (+)-pentazocine [(+)-PTZ], confer marked retinal neuroprotection in vivo and in vitro. Recently we analyzed the retinal phenotype of mice lacking σR1 (*σR1* KO) and observed normal retinal morphology and function in young mice (5–30 weeks) but diminished negative scotopic threshold responses (nSTRs), retinal ganglion cell (RGC) loss, and disruption of optic nerve axons consistent with inner retinal dysfunction by 1 year. These data led us to test the hypothesis that σR1 may be critical in forestalling chronic retinal stress; diabetes was used as the model of chronic stress.

**Methods:**

To determine whether σR1 is required for (+)-PTZ neuroprotective effects, primary RGCs isolated from wild-type (WT) and *σR1* KO mice were exposed to xanthine–xanthine oxidase (10 µM:2 mU/ml) to induce oxidative stress in the presence or absence of (+)-PTZ. Cell death was evaluated by terminal deoxynucleotidyl transferase dUTP nick end labeling (TUNEL) analysis. To assess effects of chronic stress on RGC function, diabetes was induced in 3-week C57BL/6 (WT) and *σR1* KO mice, using streptozotocin to yield four groups: WT nondiabetic (WT non-DB), WT diabetic (WT-DB), *σR1* KO non-DB, and *σR1* KO-DB. After 12 weeks of diabetes, when mice were 15-weeks old, intraocular pressure (IOP) was recorded, electrophysiologic testing was performed (including detection of nSTRs), and the number of RGCs was counted in retinal histological sections.

**Results:**

In vitro studies showed that (+)-PTZ could not prevent oxidative stress-induced death of RGCs harvested from *σR1* KO mice but afforded robust protection against death of RGCs harvested from WT mice. In the studies of chronic stress induced by diabetes, the IOP measured in the four mouse groups was within the normal range; however, there was a significant increase in the IOP of *σR1* KO-DB mice (16±0.5 mmHg) compared to the other groups tested (*σR1* KO non-DB, WT non-DB, WT-DB: ~12±0.6 mmHg). Regarding electrophysiologic testing, the nSTRs of *σR1* KO non-DB mice were similar to WT non-DB mice at 15 weeks; however, they were significantly lower in *σR1* KO-DB mice (5±1 µV) compared to the other groups, including, notably, *σR1* KO-nonDB (12±2 µV). As expected, the number of RGCs in *σR1* KO non-DB mice was similar to WT non-DB mice at 15 weeks, but under chronic stress of diabetes there were fewer RGCs in retinas of *σR1* KO-DB mice.

**Conclusions:**

This is the first report showing unequivocally that the neuroprotective effects of (+)-PTZ require σR1. *σR1* KO mice show normal retinal structure and function at young ages; however, when subjected to the chronic stress of diabetes, there is an acceleration of retinal functional deficits in *σR1* KO mice such that ganglion cell dysfunction is observed at a much earlier age than nondiabetic *σR1* KO mice. The data support the hypothesis that σR1 plays a key role in modulating retinal stress and may be an important target for retinal disease.

## Introduction

Sigma receptor 1 (σR1) is a ~27-kDa transmembrane protein originally described as an opioid receptor [1] but later identified as a unique pharmacological receptor [2]. It shares no sequence homology with any other mammalian protein. The endogenous function of σR1 is not certain; owing to its location at the endoplasmic reticulum (ER)–mitochondrial membrane, it may play an important role as a chaperone modulating ER stress [3,4]. Beneficial effects of putative σR1 ligands, such as decreased pain, enhanced memory, and neuroprotection, suggest that σR1 could be an important therapeutic target in several diseases [3], including ocular and retinal diseases. σR1 is expressed in multiple ocular tissues, such as lacrimal gland [5], cornea, iris–ciliary body, lens, and retina [6,7]. In retina it is expressed abundantly in the ganglion cell and inner nuclear layers [6,8-10], in photoreceptor [6,9] and retinal pigment epithelium (RPE) cells [6,11], and the optic nerve [6,10]. In isolated retinal Müller and retinal ganglion cells (RGCs), σR1 has been detected on ER and nuclear membranes [12,13].

Robust retinal neuroprotective effects of σR1 ligands have been reported by several laboratories; these effects include protection against RGC apoptotic death in vitro using the putative σR1 ligands (+)-pentazocine [(+)-PTZ] and SKF-10,047 [14-18]. Marked preservation of retina was observed in vivo in *Ins2^Akita/+^* diabetic mice treated for several weeks with (+)-PTZ [19]. Given its role in neuroprotection and cell survival, its abundant expression in eye, and its putative function as a molecular chaperone, we asked recently whether σR1 is critical for ocular development and/or maintenance of normal ocular structure/function. The availability of genetically manipulated mice lacking σR1 (*σR1* KO mice) [20] offered a tool to address this question. Functional, morphologic, and cell biologic tools were used to examine comprehensively the ocular phenotype in *σR1* KO versus wild-type (WT) mice over a 1-year period [21]. The data showed that the anterior segments of the eye (cornea, lens, ciliary body–iris) were histologically normal in *σR1* KO mice and intraocular pressure (IOP) was within normal limits at all ages examined. In the retina, however, there were functional and morphologic changes observed, albeit not until the mice were several months old. For example, electrophysiologic changes in *σR1* KO mice, including significantly decreased electroretinogram (ERG) b-wave amplitudes and diminished negative scotopic threshold responses (nSTRs), emerged at approximately12 months of age consistent with a late-onset inner retinal dysfunction. Morphologic analyses revealed significantly fewer cells in the ganglion cell layer, but again not until the mice were many months of age. Ultrastructural studies provided evidence of disrupted optic nerve axons, including accumulation of organelles (swollen mitochondria) and glial cell apoptosis, in 33-week-old mice.

While σR1 is not essential for normal ocular/retinal development, the reviewed results suggest it may play a critical role in forestalling retinal cellular stress. Recently, Guo and colleagues investigated this role in an acute injury model [10]. They performed intraorbital optic nerve crush in *σR1* KO mice and determined that the number of surviving cells in the ganglion cell layer of *σR1* KO was significantly decreased (18.5%) compared to WT mice subjected to the same injury. Their data strongly support the notion that lack of σR1 increases susceptibility to acute retinal injury. The effects of chronic stress to the retina in the absence of σR1, however, have not been explored. In the present study we asked whether the late-onset RGC death reported for *σR1* KO mice, which is not observed until the mice are ~1 year [21], would be accelerated under the chronic stress of diabetes. Diabetes represents one of the most clinically relevant forms of chronic stress encountered by retina; it is the leading cause of new cases of blindness among adults aged 20–44 years, causing from ~12,000 to 24,000 new cases of blindness yearly [22]. The myriad consequences of this disease on retina include neuronal cell loss and vascular complications [23]. In the present study we induced diabetes in *σR1* KO and WT mice and investigated IOP, electrophysiologic function, and RGC viability in the animals after 12 weeks of diabetes. Our data show that the chronic stress of diabetes accelerates RGC dysfunction in the *σR1* KO mice and suggest that σR1 is an important player in managing retinal stress.

## Methods

### Animals

The generation of σR1 KO mice, establishment of the colony in our animal facility, and details about genotyping have been described [21]. Briefly, the heterozygous mice were obtained from Mutant Mouse Resource Regional Center and implanted into female C57BL/6J mice (Jackson Laboratories, Bar Harbor, ME) at The Scripps Research Institute. The generation of σR1 KO mice, establishment of the colony in our animal facility, and details about genotyping have been described [21]. Briefly the genotyping uses polymerase chain reaction (PCR) to determine the presence of σR1 using three primers (a) 5′-TCT GAG TAC GT G CTG CTC TTC G-3′, (b) 5′-ATA AAC CCT CTT GCA GTT GCA TC-3′, (c) 5′-GAA ACT GCC GT G TTC TGT TTC C-3′ the conditions of which were: 30 cycles at 94 °C (15 s), 55 °C (30 s) and 72 °C (40 s). Founder heterozygous mice were transferred to the animal facility at Georgia Health Sciences University to allow us to establish our colony. For the first set of experiments in which RGCs were isolated, 78 neonatal mice (*σR1* KO and WT) were used as described below. In the studies examining the effects of diabetes, 40 mice were used to generate four mouse groups: (1) WT nondiabetic (WT non-DB), (2) WT diabetic (WT-DB), (3) *σR1* KO non-DB, and (4) *σR1* KO-DB. Mice were made diabetic at 3 weeks of age, using streptozotocin per our method [24]. Briefly, they received an intraperitoneal injection of 75 mg/kg STZ dissolved in sodium citrate buffer (0.01 M, pH 4.5) on three successive days. Mice were maintained for 12 weeks after which they were weighed, subjected to functional testing as described below, and blood glucose and insulin levels measured. Mice were not administered insulin at any time during the experiments and typically do not survive beyond approximately18–20 weeks of diabetes, which was the rationale for ending the experiment after 12 weeks diabetes duration (when mice were 15 weeks of age). Mice were maintained for 12 weeks after which they were weighed, subjected to functional testing as described below. Blood was collected from the cardiac ventricle at the time the animals were euthanized; blood samples were used immediately to determine glucose and insulin levels (described below). Mice were rapidly euthanized by carbon dioxide asphyxiation/cervical dislocation per our approved protocol. Eyes were harvested at the termination of the experiment and prepared for cryosectioning per our method in immunohistochemical studies [19,21]. Briefly, the eyes were oriented in Optimal Cutting Temperature compound (Electron Microscopy Sciences, Hatfield, PA), frozen slowly in liquid nitrogen and then cryosectioned (10 μm thickness). The sections were placed on Superfrost Slides (Fisher Scientific Corp., Pittsburgh, PA) and stored at −80 °C until used. Maintenance of animals adhered to the Georgia Health Sciences University (GHSU) institutional guidelines for the humane treatment of animals following our IACUC approved protocol and to the Association for Research in Vision and Ophthalmology (ARVO) Statement for the Use of Animals in Ophthalmic and Vision Research.

### Analysis of (+)-pentazocine as a neuroprotective ligand via sigma receptor 1

Primary RGCs were isolated from mouse pups (WT and *σR1* KO) at postnatal day 3. Immunopanning procedures and verification of purity of the cells have been described in detail [13,15]. Briefly, retinas were subjected to a two-step process using anti-macrophage antiserum to remove the macrophages and microglial cells; the non-adherent cells were then incubated in with anti-mouse Thy-1.2 antibody to isolate the ganglion cells. The purity of the cells has been verified to show that they are positive for neuronal markers and negative for glial and RPE markers. Cells were seeded at a density of 2.3×10^5^ cells per well and were incubated at 37 °C in media that was changed every 2 days. Since these are primary cultures of RGCs, they are not amenable to passage and they do not proliferate; they do, however, extend neurite processes, evincing characteristics of neurons. Differential interference contrast (DIC) images were captured using a Nikon ECLIPSE TS100 inverted microscope (Nikon, Sendai, Japan) equipped with a Moticam 2300 camera (Motic Instruments Inc., Richmond, British Columbia, Canada) and Motic Images Plus 2.0 software (Motic Instruments Inc.). To determine whether (+)-PTZ, a putative σR1 ligand, would afford protection in the absence of σR1, cells were exposed to xanthine–xanthine oxidase (X:XO 10 μM:2 mU/ml) in the presence/absence of 3 µM (+)-PTZ (Sigma-Aldrich Corp, St. Louis, MO) for 18 h. The number of cells undergoing apoptosis, as detected by the terminal deoxynucleotidyl transferase dUTP nick end labeling (TUNEL) assay, was quantified as described previously [13,15]. Exposure to X:XO is a well known method for generating superoxide and hydrogen peroxide in a molar ratio of approximately 1:3 [25]. The concentration and duration of exposure to X:XO as well as the concentration of (+)-PTZ used in this study were based on extensive previous studies showing that this level of oxidative stress significantly increases cell death and that 3 µM (+)-PTZ can significantly inhibit cell death [13,15]. Essentially, the present experiments recapitulated those studies in cells that have σR1 (RGCs from WT) versus those that lack this putative target for (+)-PTZ (RGCs from *σR1* KO).

### Measurement of intraocular pressure and retinal function

IOP was measured by placing the probe of a handheld iCare rebound tonometer (Icare Finland Oy, Espoo, Finland) on the cornea [21]. Negative scotopic threshold recordings (nSTRs) were performed under isoflurane anesthesia in diabetic and nondiabetic mice. The nSTRs were obtained with 5-ms flashes of a blue (470 nm) light emitting diode, made dimmer with neutral-density filters and defocusing. Just above threshold, a late negative potential develops at ∼200 ms after the flash; this is the negative (n)STR [21]. Daytime measurements were obtained at approximately12:00 noon and nighttime measurements at approximately12:00 midnight. Mouse weights were determined along with blood glucose, using a glucometer (Abbot Diabetes Care, Alameda, CA), and blood insulin, measured using the Ultra Sensitive Mouse Insulin ELISA Kit (Crystal Chem Inc., Downers Grove, IL) per the manufacturers’ instructions and blood insulin was measured using the Ultra Sensitive Mouse Insulin ELISA Kit (Crystal Chem Inc., Downers Grove, IL) in a spectrophotometric assay.

### Quantitative immunohistochemical analyses of cells in the ganglion cell layer

Brn3a is a well characterized marker for RGCs [26], and cleaved caspase-3 is a marker for apoptosis [21]. Immunohistochemical detection methods were used to determine the number of RGCs in retinal cryosections, using a goat polyclonal antibody against Brn-3a (1:100; Santa Cruz Corp., Santa Cruz, CA) followed by incubation with AlexaFluor-488-conjugated donkey anti-goat immunoglobulin (IgG) secondary antibody (1:1,000; Invitrogen, Carlsbad, CA). To detect apoptotic cells, sections were incubated with a rabbit polyclonal antibody against cleaved caspase-3 (1:250; Cell Signaling Technology, Beverly, MA). For detection of immunopositive signals, retinal sections were incubated with AlexaFluor-555-conjugated donkey anti-rabbit IgG secondary antibody (1:1,000; Invitrogen). Coverslips were mounted on slides using fluorescein with 4',6-diamidino-2-phenylindole (DAPI; Sigma-Aldrich Corp) to label nuclei and viewed by epifluorescence, using a Zeiss Axioplan-2 microscope (Carl Zeiss, Oberkochen, Germany) equipped with the AxioVision program (version 4.6.3) and an high resolution microscopy (HRM) camera. Brn3a-positive cells were counted, and data were expressed as the number of cells per 100 µm retinal length. Slides incubated with only the secondary antibody were used as negative control; there was no labeling detected in the absence of the primary antibody (data not shown).

### Statistical analysis

The data for ERG analysis and quantification of RGCs in retinal sections were analyzed by the Student *t* test. One-way ANOVA was used to determine whether there were significant differences in the number of TUNEL-positive cultured RGCs, bodyweight, blood glucose levels, insulin levels, and IOP. Tukey’s paired comparison test was the post hoc statistical test. Statistical analysis was conducted using the GraphPad Prism analytical program (GraphPad Software, Inc., San Diego, CA). A p value of <0.05 was considered significant.

## Results

### Sigma receptor 1 is required for (+)-pentazocine neuroprotection against oxidative-induced stress

**Figure 1:**
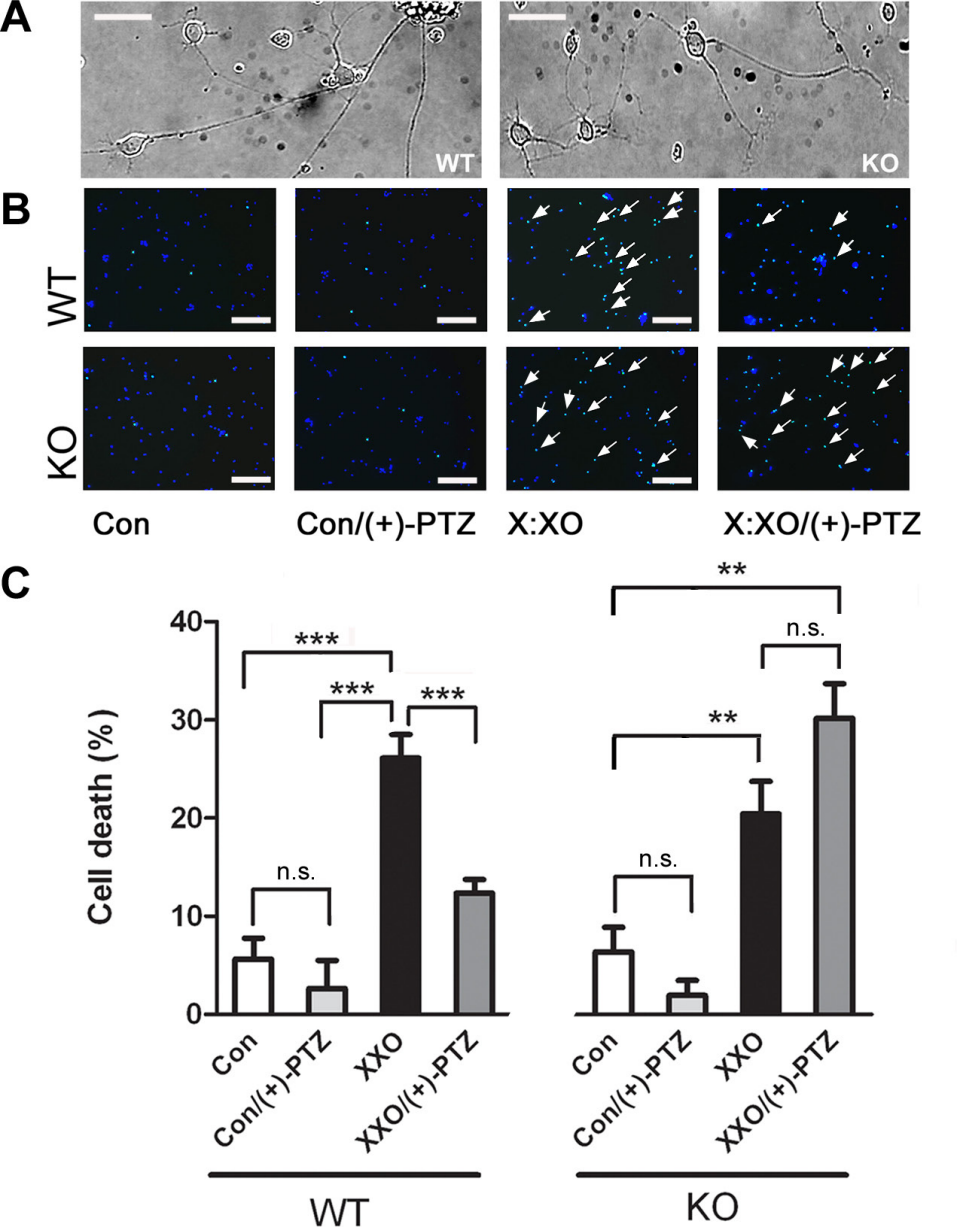
Sigma receptor 1 is required for (+)-PTZ-mediated neuroprotection. RGCs were isolated from neonatal WT and *σR1* KO mouse pups and cultured for 72 h. **A**: DIC images were captured of living cells. Subsequently, cells were treated with xanthine–xanthine oxidase (X:XO; 10 µM:2 mU/ml) in the presence or absence of (+)-PTZ (3 µM) for 18 h and were subjected to TUNEL analysis. The calibration bar=10 μm. **B**: Representative photomicrographs of data from the TUNEL assay in cells receiving no treatment, (+)-PTZ only, X:XO only, or X:XO and (+)-PTZ. Nuclei, labeled with DAPI, fluoresce blue, and TUNEL-positive cells fluoresce green (arrows). The calibration bar=50 μm. **C**: Quantification of TUNEL-positive cells; data collected from 9 fields for each treatment; experiments were repeated three times. Data are expressed as mean±SEM of the ratio of apoptotic cells to the total number of cells. (The asterisks denote that data are significantly different from control; **, p<0.01, ***, p<0.001).

There have been several studies suggesting that (+)-PTZ is protective against neuronal cell death; however, it is not known whether (+)-PTZ requires σR1 to confer neuroprotection or whether it might act through some other receptor. The availability of *σR1* KO mice allowed this question to be addressed. During the early postnatal period, retinal function and structure appear normal in *σR1* KO mice [21], permitting isolation and culture of RGCs from these mutant mice. RGCs isolated from WT and *σR1* KO mice were used to examine whether (+)-PTZ would afford neuroprotection in the absence of σR1, its putative target. RGCs were isolated from WT and *σR1* KO neonatal mice and allowed to grow in culture media for 72 h, over which time the extension of neurite processes was examined. Differential interference contrast microscopy (DIC) microscopy revealed neurite development in RGCs harvested from *σR1* KO mice as well as WT (Figure 1A). The dendritic arborizations and axonal projections were comparable between the two mouse groups. When cells were treated with X:XO, there was a significant increase in the number of TUNEL-positive RGCs in WT and *σR1* KO RGCs; approximately 20%–25% of RGCs died within 18 h incubation with X:XO (Figure 1B). Thus, RGCs of *σR1* KO mice are susceptible to acute oxidative stress in a manner similar to WT. When the X:XO-exposed cells harvested from WT mice were co-treated with (+)-PTZ, there was a marked decrease in cell death, which is consistent with previous reports [13]. When RGCs harvested from *σR1* KO mice were exposed to X:XO in the presence of (+)-PTZ, however, there was no protection against cell death. There was no significant difference in cell death observed in the X:XO treated group compared to the X:XO (+)-PTZ group. From these experiments we conclude that (+)-PTZ mediates neuroprotection by interacting with σR1 and that σR1 is obligatory for (+)-PTZ to be neuroprotective against RGC death.

### Bodyweight, blood glucose, and insulin levels in diabetic mice versus controls

**Figure 2:**
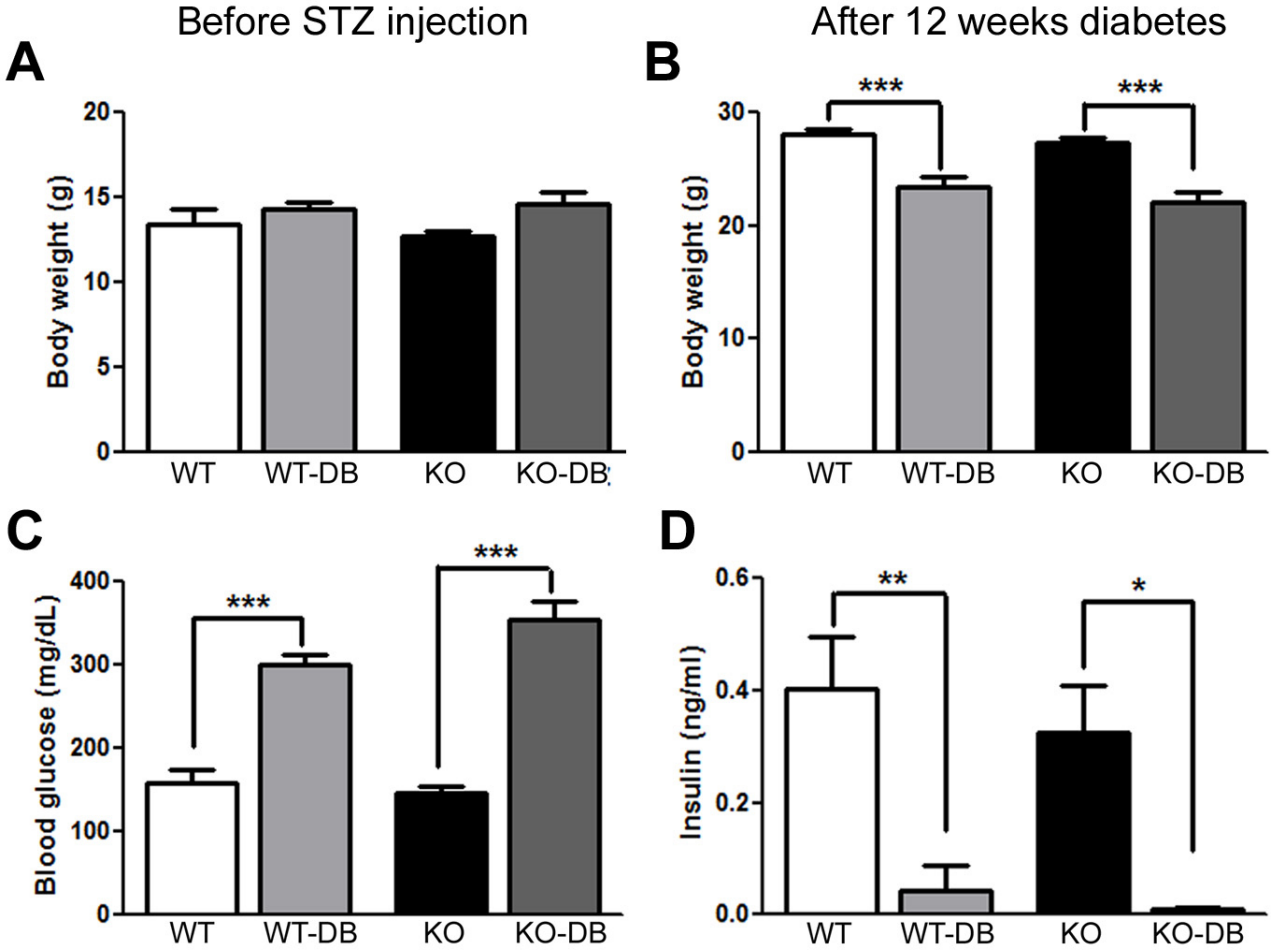
Bodyweight, blood glucose and insulin levels. Wild-type and σR1 KO mice were made diabetic using streptozotocin (STZ) at 3 weeks and the bodyweights and insulin levels determined from blood samples harvested at euthanasia. **A**: Bodyweights are provided for mice before STZ injection; **B**: Bodyweights are provided for mice at the termination of the experiment (12 weeks following STZ injection); **C**: Blood glucose levels are provided for mice 12 weeks following STZ injection; and **D**: Insulin levels are provided for mice 12 weeks following STZ injection. For these studies, 10 mice were tested per group (n=10). The data were significant as shown by the asterisk representing statistical analysis. (The asterisks denote between which groups there are significant statistical differences: *, p<0.05; **, p<0.01; ***, p<0.001).

To determine whether in vivo chronic stress, in the form of diabetes, altered retinal structure and function in *σR1* KO mice and in particular accelerated disruption of the retina, streptozotocin was administered to WT and *σR1* KO mice. Bodyweight was determined before the streptozotocin injection and again at the termination of the experiment. Blood glucose levels and insulin levels were determined at the termination of the experiment. There were no differences in the weights of mice before induction of diabetes in any of the mouse groups analyzed (Figure 2A). After 12 weeks of diabetes, WT-DB and *σR1* KO-DB mice weighed significantly less than WT non-DB and *σR1* KO non-DB mice (Figure 2B). Blood glucose levels were elevated significantly in the WT-DB and *σR1* KO-DB mice compared to their respective nondiabetic controls (Figure 2C); blood insulin levels were markedly reduced in the WT-DB and *σR1* KO-DB mice (Figure 2D). There was a trend toward higher blood glucose levels and lower insulin levels in the *σR1* KO*-*DB versus WT-DB, although the data did not reach statistical significance.

### Increased intraocular pressure in diabetic *σR1* KO mice

**Figure 3:**
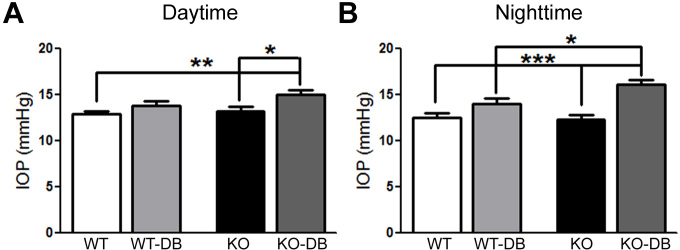
Intraocular pressure. Intraocular pressure (IOP) was measured in wild-type, non-Diabetic (WT-DB), Wild-type-diabetic (WT-DB), σR1 knockout non-diabetic (σR1 KO non-DB), and σR1 knockout diabetic (σR1 KO-DB) mice during the day (**A**) and night (**B**) after 12 weeks of diabetes. Data represent averaged values for the mice in the four groups (n=10 mice tested per group; the asterisks denote between which groups there are significant statistical differences: *, p<0.05; **, p<0.01; ***, p<0.001).

In our initial characterization of the retinal phenotype of *σR1* KO mice, we measured IOP but did not observe any differences in this parameter over the 1-year period studied [21]—that is, lack of σR1 alone does not increase IOP. We wanted to investigate whether the additional stress of diabetes would alter IOP in the absence of σR1. IOP was examined after 10–12 weeks duration of diabetes. Diurnal variations in IOP have been reported [27]. The IOP measured for all animals was within the range of 10–20 mmHg, which is within normal limits. It is noteworthy that although within the normal range, the IOP in *σR1* KO-DB mice was significantly higher than for any other mouse group studied. It was significantly higher than *σR1* KO non-DB during the day (15.0±0.5 versus 13.25±0.46 mmHg, respectively, Figure 3A) as well as at night (16.2±0.5 mmHg versus 12.35±0.6, respectively, Figure 3B), in addition to being higher than WT non-DB and WT-DB mice at night. These data suggest that while diabetes itself is insufficient to increase IOP, it tends to increase IOP when σR1 is absent.

### Decreased scotopic threshold responses in diabetic *σR1* KO mice

**Figure 4:**
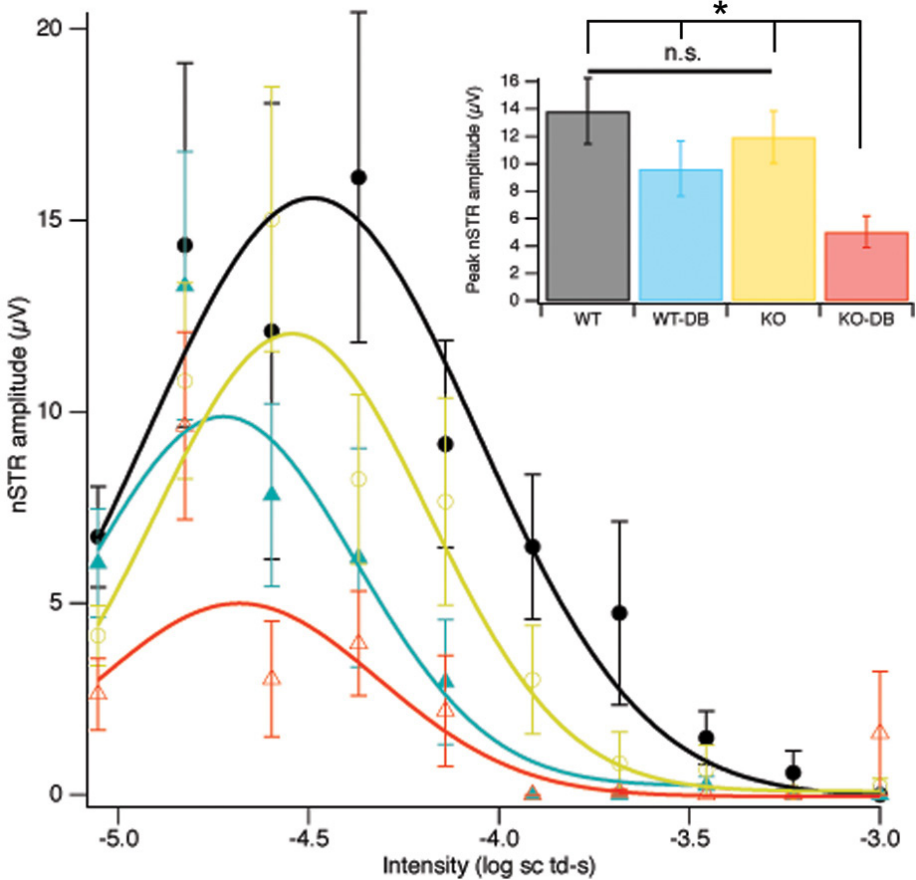
Negative scotopic threshold responses. Electrophysiologic analysis was performed after 12 weeks of diabetes. Under scotopic conditions, responses to a range of dim flash intensities were recorded. Means and standard errors averaged over 10 mice per group are shown across intensities given in log scotopic troland-seconds. Gaussian fits to the results for each group are shown. The peaks, and their estimated standard errors, of these Gaussians are compared in the inset, with the significance (*t* test *, p<0.05) of the differences indicated.

STRs are the most sensitive ERG responses observable with dim stimuli in the dark-adapted state and are a reflection of RGC health [28,29]. They are obtained with 5 ms flashes of a blue (470 nm) light-emitting diode, made dimmer with neutral density filters and defocusing. Just above threshold, a late negative potential develops at approximately 200 ms after the flash; this is the nSTR. One-year-old *σR1* KO mice have diminished nSTRs compared to WT mice [21], reflecting late-onset inner retinal dysfunction. In the present study, nSTRs were recorded in mice after 12 weeks of diabetes (~15 weeks of age). While there was no significant difference between nSTRs of *σR1* KO non-DB (12±2 µV) versus WT non-DB (14±2 µV) at this age (Figure 4), there was a significant reduction in nSTRs in the *σR1* KO-DB mice (5±1 µV). There was a significant reduction also in nSTRs of the *σR1* KO-DB mice compared to WT-DB mice (10±2 µV). These data suggest that the decline in RGC function characteristic of 1-year-old *σR1* KO mice is differentially accelerated by diabetes in the *σR1* null mutants.

### Assessment of retinal ganglion cells in diabetic *σR1* KO mice

**Figure 5:**
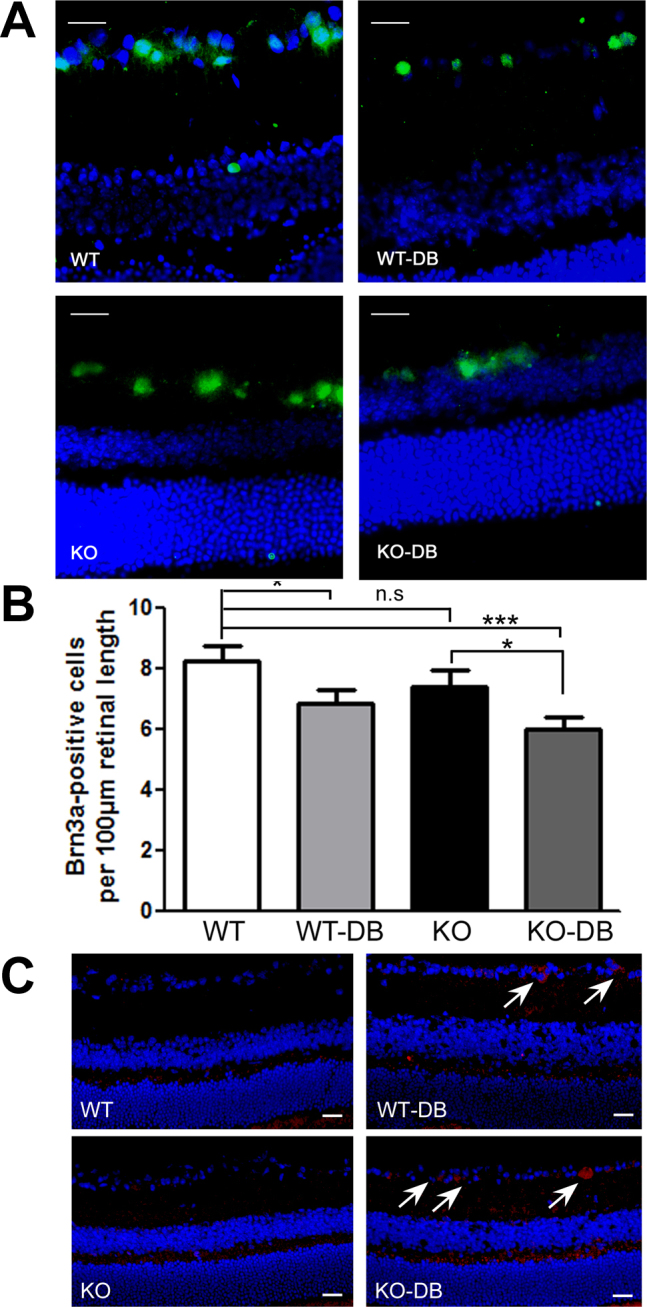
Assessment of retinal ganglion cells (RGCs) in σR1 knockout (σR1-KO) and wild-type (WT) diabetic (DB) and non-diabetic mice (non-DB). Retinal cryosections were prepared from WT (non-DB), WT-DB, σR1 KO (non-DB), and σR1 KO–DB mice after 12 weeks of diabetes and subjected to immunohistochemistry. **A**: RGCs were labeled with Brn3a (green fluorescence) and nuclei were labeled with DAPI (blue). **B**: The numbers of Brn3a positive cells were counted and expressed per unit length of retina. **C**: Red and blue fluorescent signals reflect cleaved caspase-3 and DAPI staining of nuclei, respectively. The calibration bar in panels **A** and **C**=20 μm. Statistical differences between groups is denoted with an asterisk (*, p<0.05).

RGC loss has been reported in retinas of *σR1* KO mice but only in mice of advanced age (~1 year); the numbers of RGCs in the 5 and 18 week *σR1* KO mice were not significantly different from age-matched WT mice [21]. To determine whether diabetes accelerated the loss of RGCs in the absence of σR1, immunohistochemical methods were used to label these cells in retinal cryosections (Figure 5A); the Brn3a-positive cells were then counted and expressed as number of cells in the ganglion cell layer (Figure 5B). There are several important observations from the data. There were significantly fewer RGCs in *σR1* KO-DB mice compared with WT mice. That is, there is a marked decrease in the number of RGCs when diabetes occurs in the absence of σR1. Additionally, there were significantly fewer RGCs in *σR1* KO-DB compared to σR1 (non-DB) as well as in the WT-DB compared to WT (non-DB) mice. The difference in the two deltas (WT group–DB versus non-DB compared to *σR1* KO–DB versus nondiabetic) did not reach statistical significance. It is likely that frank dysfunction of the ganglion cells as measured in the electrophysiologic studies precedes measureable loss of the cells. We investigated also whether cells were undergoing apoptosis as evidenced by expression of cleaved caspase-3. It is clear that in both the *σR1* KO-DB and the WT-DB mice there are more caspase-3- positive cells (Figure 5C), whereas caspase-3-positive cells were rarely observed in the WT or the *σR1* KO mice at the age studied.

## Discussion

There were two major findings of the present study. The first was that the neuroprotective functions of (+)-PTZ are mediated via σR1, and the second was that in the absence of σR1, chronic stress accelerates RGC dysfunction.

Regarding the first finding, we took advantage of the observation that the absence of σR1 does not hinder normal retinal development [21] to investigate whether the previously reported neuroprotective effects of (+)-PTZ are mediated via σR1 versus another mechanism (e.g., a different receptor). While (+)-PTZ is considered a highly specific ligand for σR1 with an affinity in the nanomolar range (0.0046 µM [K_d_]) [30], it has not been demonstrated unequivocally that (+)-PTZ mediates neuroprotection via σR1. Moreover, there have been reports of alternative targets for (+)-PTZ [31]. Thus, we designed an experiment to examine this question specifically in ganglion cells, the neuron that is particularly vulnerable in diabetic retinopathy [23]. By isolating ganglion cells from WT mice and from *σR1* KO mice, we had a neuronal population that either did or did not contain σR1. The cells could be manipulated using a known stressor and we had clear endpoints (neurite processes, TUNEL positivity) that could be analyzed in a straightforward manner to inform whether (+)-PTZ afforded protection. RGCs were isolated following a well established immunopanning procedure from neonatal WT and *σR1* KO mice; they were cultured under oxidative stress conditions with or without (+)-PTZ. The data showed that RGCs from *σR1* KO mice succumbed to oxidative stress in a manner similar to those harvested from WT; however, unlike WT-treated cells, (+)-PTZ did not prevent death in cells isolated from *σR1* KO mice. That is, (+)-PTZ did not protect against oxidative stress in cells lacking σR1. These findings provide compelling evidence that neuroprotective effects of (+)-PTZ are obligatorily dependent on σR1. The rationale for inducing neuronal death using oxidative stress is that oxidative stress (the overproduction of reactive oxygen species) has been measured indirectly in patients. Data suggest that reactive oxygen species levels are related to the severity of diabetic complications, including diabetic retinopathy [32-35]. It is noteworthy also that xanthine oxidase is increased in type 1 diabetes [36]. As an extension of the in vitro studies reported here, future studies using *σR1* KO mice could be performed in which a known retinal stressor is used to induce retinopathy in the presence/absence of (+)-PTZ to validate the findings we have obtained using the isolated cell approach.

Regarding the second finding of accelerated RGC dysfunction under chronic stress, σR1 is an abundantly expressed protein whose endogenous role in cells has been elusive. The present study examined the potential role of σR1 as a stress modulator in retina. Earlier studies provided compelling evidence that ligands for σR1 afford robust retinal neuroprotection. This has been demonstrated in vivo and in vitro in several independent laboratories. For example, Techedre and colleagues showed that the σR1 ligand SKF-10,047 could protect against RGC death by regulating intracellular calcium as well regulating expression of key apoptosis genes [37]. Bucolo and coworkers demonstrated that pretreatment with PRE-084, a selective σR1 agonist, increased viability of RPE cells and decreased DNA damage induced by oxidative insult [16]. Our laboratory has demonstrated protective effects of (+)-PTZ against oxidative and excitotoxic RGC death in vitro [13-15] and RGC death associated with diabetes in vivo [19].

Collectively, these findings suggest an important neuroprotective role of σR1 in retina. Interestingly, recent comprehensive studies of the retinal structure and function of *σR1* KO mice demonstrate that σR1 is not required for normal retinal development [10,21]. Indeed, while *σR1* KO mice demonstrate a late-onset retinal degeneration characterized by loss of RGCs, ultrastructural alterations of the optic nerve, and diminished ERG (nSTRs), the phenotype is not observed until the mice are nearly 1 year of age [21]. These observations led us and others to speculate that σR1 may play a role in modulating stress in the retina. Mavlyutov and colleagues investigated this using optic nerve crush in WT and *σR1* KO mice to determine whether lack of σR1 rendered animals more susceptible to acute injury. Their data showed that 7 days after the crush, WT mice retained ~90% of their optic nerve axons while *σR1* KO mice retained only ~70% [10]. They concluded that σR1 delays crush-induced RGC degeneration and that RGC death increases under acute injury in the absence of σR1.

In the current work, we examined the role of σR1 under chronic retinal stress in the form of diabetes. Diabetes was induced in WT mice and those lacking σR1 by injecting streptozotocin at 3 weeks of age. Eyes were evaluated 12 weeks post onset of diabetes when mice were 15 weeks of age. When *σR1* KO non-DB mice were analyzed at this age, no functional deficits or structural alterations were observed, confirming earlier findings [21]. However, rendering the *σR1* KO mice diabetic accelerated retinal dysfunction. Retinas were examined functionally by assessing IOP and nSTRs. *σR1* KO-DB mice had IOPs that were significantly elevated at night compared to *σR1* KO non-DB mice as well as to WT non-DB and WT-DB mice. The levels detected were ~15 mmHg, which is within the normal range; nevertheless, the elevation in *σR1* KO-DB was significantly greater than in the other mouse groups examined. Whether σR1 plays a role in modulating IOP would be a fruitful area for further study. For example, it would be interesting to explore other chronic stressors in *σR1* KO mice for their propensity to elevate IOP. Of note, Buculo reported that topical σR1 agonists lower IOP in a rabbit model [38].

The other functional test performed was measurement of nSTRs; our data showed a marked decrease in nSTRs of the *σR1* KO-DB mice compared to the other mice in the study. The nSTRs ranged between 9 and 13 µV for WT non-DB, WT-DB, and *σR1* KO non-DB mice compared to ~5 µV in the *σR1* KO-DB mice. The nSTR is a highly sensitive test for RGC activity; thus, these data provide strong evidence that σR1 modulates RGC function under chronic stress. As is the case with acute stress [10], chronic stress can accelerate RGC dysfunction in the absence of σR1. Accompanying the decreased RGC function was a decrease in the numbers of Brn3a-positive cells detected in the ganglion cell layer of *σR1* KO-DB mice compared with WT mice. Our data clearly show that there is a much earlier loss of RGCs and evidence of inner retinal dysfunction in *σR1* KO-DB mice compared with WT mice; this supports the role of σR1 in forestalling retinal stress. There is a decrease in the number of RGCs in *σR1* KO-DB compared to σR1 (nondiabetic), although the decrease is similar to the decrease in cell number between WT and WT-DB. It appears that cell loss as an endpoint is not as severe an indicator as the nSTR and IOP changes we observed.

In summary, the in vivo data comparing diabetic versus nondiabetic *σR1* KO mice have allowed us to investigate the role of chronic stress on retinal function in the absence of σR1. The acceleration of ganglion cell dysfunction during chronic diabetic stress coupled with the late onset inner retinal dysfunction of nondiabetic *σR1* KO mice underscores the role of this protein as a stress modulator. These data complement the findings of the Guo laboratory [10] showing the importance of σR1 in an acute injury model. Collectively, the findings set the stage to determine the mechanism by which σR1 mediates neuroprotection in retina.
